# Sex-related Differences of *Excoecaria agallocha* L. with a View to Defence and Growth

**DOI:** 10.21315/tlsr2022.33.2.4

**Published:** 2022-07-15

**Authors:** Abdul Kader, Sankar Narayan Sinha

**Affiliations:** Department of Botany, University of Kalyani, Nadia, West Bengal 741235, India

**Keywords:** Biological Activities, *Excoecaria agallocha*, Mangrove, Sex-related Response, The Sundarbans, Specific Leaf Area, Total Phenolics Estimation

## Abstract

*Excoecaria agallocha* is a dioecious mangrove species, reported to have various medicinal properties. This study compares the gender-related biological activities between the sexes of *E. agallocha* in accordance with morpho-biochemical parameters which indicate their fitness over various environmental stresses as well as some information about the investment of their resources over defence and growth. For this investigation, mature sun leaves of *E. agallocha* were collected from each sex from three different ecological regions like Bokkhali, Jharkhali, and Kolkata, West Bengal. The comparative study found that leaf of female plants yielded more compounds than males and also resulted in higher anti-bacterial, reducing power, total antioxidant, and lipid peroxidation activities. Female leaves also showed higher concentrations of biochemical constituents like chlorophyll a, chlorophyll b, total carotenoids, total phenolic compounds and total protein content than male leaves. However, leaf area of all-male leaves in all sites was found to be greater than female leaves. The differences in growth rate and secondary metabolite content-based defences between sexes suggest that females invest resources in defences or resistance (Relative electrolytic leakages and TBARS content), whereas male invests their resources in growth (Leaf area) or tolerance. The present study strongly suggests that the female plant’s yields are more beneficial in terms of medicinal properties as compared to the male plant.

**Highlights**

- The antibacterial activity, reducing power and total antioxidant activity of leaf crude extracts of female species of *Excoecaria agallocha* were higher than male leaf extracts. This finding was endorsed by higher phenolic content in female leaves than in the males of *E. agallocha*.
- The female species of *Excoecaria agallocha* respond more to different stresses than the male species by increasing the concentration of chlorophylls, carotenoids, and proteins in their leaves. The differences in growth rate (Leaf area) and secondary metabolite content-based defenses between the two sexes suggest that females invest resources in defenses or resistance whereas male invests their resources in growth (Leaf area) or tolerance (Based on relative electrolytic leakages and TBARS content).
- This study suggested that the plantation of the female of this species is more beneficial than males according to several medicinal properties.

## INTRODUCTION

The plant evolution mainly depends on its competitive ability to overcome various environmental pressures (Coley *et al*. 1985). In this context, plants have modified their defence mechanism including variations in the chemical arrangement of structures, the comparative mass of different assemblies or organs and the relative numbers of diverse structures (Bazzaz *et al*. 1987). Recently, researchers have a keen interest in finding the differences between the sexes of dioecious plants in response to various natural or artificial environmental stresses in a number of plant species. They differentiated male and female individuals on the basis of many morphological and physiological as well as ecological factors in a number of dioecious plant species (Li *et al*. 2007; Letts *et al*. 2008; Chen *et al*. 2010; Yang *et al*. 2011; Gupta *et al*. 2012).

The Indian Sundarbans is located between 21°31′–22°30′N and 88°10′–89°51′E of North and South 24-Parganas district of the West Bengal state (Naskar & Mandal 1999). This coastal deltaic ecosystem harbours many mangrove species in a variety of physiological characters and biochemical constituents (Kader & Sinha 2018). Normally, mangroves are adapted to various conditions like constant change in salinity and pH, experiences various tidal storms and other unexpected environmental situations. Nowadays, many secondary metabolites have been categorised from mangrove plants which act as own defence against various environmental stresses and possess many biological as well as medicinal importance (Patra & Thatoi 2011). *Excoecaria agallocha* L. (Euphorbiaceae) is a dioecious economically important widespread and common true mangrove tree that grows in the Sundarbans and other coastal mangrove areas in the wet tropics. The plants of *E. agallocha* have been reported for their antimicrobial, antioxidant, antiviral, antifungal and anticancer activities (Pattanaik *et al*. 2008; Patra *et al*. 2009; Simlai & Roy 2013). Besides that, mangrove forests are mostly essential for ecosystem services and biodiversity. They are the main backbone of seaside and offshore food webs. They also function as nursery estates and breeding locations for commercially and ecologically important animals, long-lasting storage for carbon, a renewable source of plant products, safeguard against coastal threats and are consequently advantageous for residential economies in almost 123 countries/territories (Carter *et al*. 2015; Wang *et al*. 2020). However, in the last few decades mangrove destruction has been noticed due to various environmental and anthropogenic activities (Kader *et al*. 2016). The main reasons behind that the conversion of mangrove habitat into cultivated areas for extension of agriculture and aquaculture, over-exploitation of forests, rapid urbanisation like construction of industries and upstream dams, eutrophication of overlying water etc. (Romañach *et al*. 2018; Rudianto *et al*. 2020).

The foundation of the mangrove ecosystem is the Indo-Malaysian area as the mangroves were first settled here and spread consequently to other tropical and the subtropical world (Alongi 2009). The largest extent of mangroves is found in Asia (42%), followed by Africa (20%), North and Central America (15%), Oceania (12%), and South America (11%). Approximately 75% of mangroves are concentrated in just 15 countries (Giri *et al*. 2011). Recently, it was estimated that at least 35% of mangrove habitat was degraded worldwide during the 1980s and 1990s (Valiela *et al*. 2001). Spalding *et al*. (1997) reported that mangroves occupy 18,100,000 ha worldwide, however, according to Giri *et al*. (2011) this estimate was revised downward to 13,776,000 ha globally, and then to 8,349,500 ha by Hamilton & Casey (2016). According to trend evaluation 15.2 million hectares of mangroves are assessed to exist worldwide as of 2005, down from 18.8 million hectares in 1980 (FAO 2007). The *E. agallocha* is usually considered as important reforestation species because of its fast-growing capacity and superb adaptation to stressful environments. To recreate mangrove forests in different degraded areas with this reforestation species, from natural inhabitants the *E. agallocha* are normally transplanted. Literature studies found that the previous research activities on *E. agallocha* were mostly focused on its conservation strategies, genetics, physiological and stress-related studies and medicinal properties. Briefly, Eganathan *et al*. (2000) propagated *E. agallocha in vivo* using cuttings and the air layering method. They found that October was best followed by January for the circulation of cuttings and induction of air layers. The maximum rooting was shown when the cuttings and air layers were formulated with indole-3-butyric acid (IBA) alone up to 2500 ppm. Another study of *in vivo* conservation exhibited that different water parameters and sedimentological characters could affect the growth and establishment of this species (Pillai & Harilal 2018). Apart from this, Rao *et al*. (1998) developed an *in vitro* propagation protocol for *E. agallocha* on a newly formulated medium. They found that benzyladenine (BA), Zeatin and IBA had a tremendous role on shoot formation whereas rooting was achieved with IBA alone on a newly formulated medium. Arumugam and Panneerselvam (2012) also developed an *in vitro* conservation strategy for this species with Modified Murashige and Skoog (MMS) medium. They showed that BA and Naphthaleneaceticacid (NAA) was better for shoot induction whereas Indoleacetic acid (IAA) alone was best for root induction.

Das *et al*. (2011) evaluated the genetic diversity in five male populations of *E. agallocha* with chromosome morphology and RAPD (Random amplified polymorphic DNA) and ISSR (Inter simple sequence repeat) markers. The cluster analysis of RAPD and ISSR profiles exhibited prominent patterns of inter-population relationships among them. However, Karyotype analysis could not differentiate five male populations. Dasgupta *et al*. (2018) also performed the genetic polymorphism of *E. agallocha* using RAPD and ISSR markers. They exhibited that relatively higher genetic polymorphism was recorded for *E. agallocha* 24.66% in RAPD; 24.87% in ISSR analysis, respectively. Yan and Tam (2013) investigated the responses of *E. agallocha* to different levels of Pb stresses. They displayed that Pb stress posed higher toxic effects on root than the leaf. The effects of salinity on seed germination and growth of young seedlings of *E. agallocha* were investigated by Chen and Ye (2014). They found that seed germination was best at 0 and 5 psu salinity. Young seedlings also performed best at 0 and 5 psu. Mariam *et al*. (2020) studied protein and proline content of early (6 to 9 months) and grown-up ages (30 months) of *E. agallocha* seedlings. They exhibited that *E. agallocha* showed the highest protein content when grown in a mesohaline zone. But at the grown-up age, this species showed the lowest content of protein. Sofia and Merlee Teresa (2016) investigated the DPPH radical scavenging activity of *E. agallocha*. They reported that methanolic extract showed maximum free radical scavenging activity in leaf samples and stem showed minimum in chloroform extract. Sultana *et al*. (2019) examined the antibacterial activities of *E. agallocha* extracts against *Pseudomonas aeruginosa*, *Staphylococcus aureus*, and *Escherichia coli*. Among all microorganisms studied, *P. aeruginosa* showed significant growth inhibition with ethanol and DMSO extracts of this mangrove species. The antimicrobial activity of *E. agallocha* against pathogenic bacteria *Escherichia coli* and *Streptococcus agalactiae* was also reported by Razzak *et al*. (2019). They exhibited that *E. agallocha* showed bactericidal activity against *E. coli* and bacteriostatic activity against *S. agalactiae*. However, there is no report on which gender has much biological activities (medicinal properties) as well as biochemical constituents or fitness over the natural environmental condition.

This study also investigates the information about the investment of their resources in defence and growth. Therefore, the present investigation formulated with the following objectives: (1) to determine which gender has much biological activities in relation to antibacterial activity, reducing power, lipid peroxidation activity and total antioxidant activities which indicates their fitness or defence over mangrove ecosystem, (2) to estimate and differentiate their biochemical parameters like phenol content, chlorophyll and carotenoid content and total protein content, (3) to differentiate sex-related differences in leaf morphological and physiological responses in normal habituate environment.

## MATERIALS AND METHODS

### Study Site

The mature sun leaves of *E. agallocha* were collected from adult trees from three individuals of each sex (Containing reproductive structures) from three different ecological regions viz., Bokkhali, Jharkhali and another from the Indian Statistical Institute, Kolkata. Soil characteristics, climatic conditions, latitude and longitude, and short general description of these sites are presented in Table 1. The distance between each sex in each site varied within ten meters. All the biological activities were conducted with leaves of respective sexes.

### Determination of pH and Electrical Conductivity (EC) of Soils

Soil samples were collected from each site at a depth of 15 cm in polythene bags and brought to the laboratory immediately after collection. The soils were air-dried, crushed using a mortar and pestle then pH and electrical conductivity (EC) were determined in 1:5 soil to distilled water suspension.

### Preparation and Extraction of Crude Material

The tap water-washed leaf samples were dried in an incubator at 40°C for 15 min to remove any traces of water. Then the leaves were air-dried for 12 weeks till constant weight was achieved. After that dried sample was ground into a 2 mm diameter powdered form. The powdered sample was kept in black plastic bags and stored in an airtight container for further work. The dried and powdered leaves (10 g) were extracted by Cold Maceration process using methanol (1:10 w/v) for 72 h. The extracts were filtered using Whatman No. 42 filter paper and then concentrated in a vacuum at 40°C using a rotary evaporator. The resulting crude extract was lyophilised and kept in the dark in a refrigerator at 4°C until tested. The extraction was done at least three times for each leaves to calculate the mean values of extractive yield.

### Measurement of Extraction Yield

The yield (on the basis of dry weight) of evaporated dried extracts was calculated by employing the following formula:


$$\text{Yield }(\%)=\frac{\text{W}1\times 100}{\text{W}2}$$

where, W1 = Weight of the extract after evaporation of the solvent and W2 = Weight of the plant sample.

### Antibacterial Activity

The standard bacterial cultures both the Gram-positive and Gram-negative used in this study were obtained from Institute of Microbial Technology, Chandigarh, India (Microbial Type Culture Collection) and from Jadavpur University (Clinical isolates), Kolkata, West Bengal, India. The Gram-negative bacterial cultures were *Salmonella typhi* (Jadavpur University), *Salmonella enterica* (MTCC 3224) and *Pseudomonas aeruginosa* (MTCC 8076) whereas the Gram-positive bacterial cultures were *Bacillus cereus* (MTCC 6629), *Serratia marcescens* (MTCC *97) and *Staphylococcus aureus* (Jadavpur University). All the bacterial strains were grown in the nutrient broth and maintained on Nutrient Agar (NA) medium (pH 7.0) slants at 4°C. The 18 h old bacterial culture was standardised using the McFarland standard (10^6^ cfu/mL of 0.5 McFarland Standard) for this study. The antimicrobial test was performed using the agar well diffusion method (Sinha & Paul 2015). To each well, 1.5 mg/mL concentrations of leaf extracts were added for each sex and incubated aerobically at 36°C and examined for any zone of inhibition after 24 h. The experiment was repeated three times and, the average values of inhibition zone diameter along with standard error were recorded.

### Reducing Power

The reducing power of aqueous plant extract was determined by the method of Oyaizu (1986). Different concentrations (50 μg/L–150 μg/L) of plant extract were mixed with potassium ferricyanide (2.5 mL, 1%). The mixture was then incubated at 50°C for 20 min. Thereafter, aliquots of trichloroacetic acid (2.5 mL, 10%) were added to the mixture, which was then centrifuged for 10 min at 2000 rpm. The upper layer of solution (2.5 mL) was mixed with ferric chloride (0.5 mL, 0.1%) solution and the absorbance was measured at 700 nm using a spectrophotometer. Control was prepared in a similar manner excluding samples. Ascorbic acids at various concentrations (50 μg/L–150 μg/L) were used as the standard.

### Total Antioxidant Activity

The phosphomolybdate method has been used to evaluate the antioxidant capacity of the extracts using ascorbic acid as the standard (Prieto *et al*. 1999). An aliquot of 0.1 mL of the methanol extract (50 μg/L–250 μg/L) was mixed with 1 mL of reagent (0.6 M sulphuric acid, 28 mM sodium phosphate and 4 mM ammonium molybdate). The tubes were capped with aluminium foil and kept in a boiling water bath at 95°C for 90 min. The absorbance of the sample was measured at 695 nm after cooling the sample at room temperature against pure methanol as a blank. IC means inhibition concentration and IC_50_ is the concentration required to result in a 50% antioxidant activity.

### Determination of Total Phenolics

The total phenolics were determined by the spectrophotometric method (Kim *et al*. 2003). Different concentrations (20 μg/L–160 μg/L) of diluted methanol extracts were added to 9 mL of distilled water. Thereafter, 1 mL of Folin-Ciocalteu’s phenol reagent was added to the mixture and shaken properly. After 5 min, 10 mL of 2% Na_2_CO_3_ solution was added to the solution. Then the mixed solution was diluted up to 25 mL with water and thoroughly mixed. After 90 min at 23°C, the absorbance was read at 750 nm. The standard curve for total phenolics was made using gallic acid standard solution (0 μg/L–160 μg/L) under the same procedure as mentioned earlier. The total phenolics were expressed as milligrams of gallic acid equivalents (GAE) per g of dried sample.

### Estimation of Total Protein

Fresh leaves (0.5 g) were homogenised with 1 mL of 50 mM sodium phosphate buffer (pH 7.0). The extracts were centrifuged at 17000 rpm for 10 min, and the supernatant was used for the determination of protein by adding 5 mL of Bradford Reagent (Bradford 1976). The absorbance was recorded spectroscopically at 660 nm using bovine serum albumin as a standard (0 μg/L–100 μg/L).

### Chlorophyll-a, b- and Total Carotenoids Estimation

Chlorophyll and carotenoid estimation were performed according to Lichtenthaler (1987) method. 10 mL of fresh leaves were grounded with liquid nitrogen. Thereafter, 8 mL of 95% ethanol was added and vortexed vigorously. The entire material was covered with aluminium foil and incubated at room temperature for overnight. Next day the samples were vortexed and centrifuged at 8000 rpm for 10 min. Chlorophyll-a, chlorophyll-b and carotenoid estimation absorbance were determined at 664.2 nm, 648.6 nm, and 470 nm, respectively. The chlorophylls and carotenoid content were expressed as mg/g fresh weight (FW) using the following formula of Lichtenthaler (1987):


$$\begin{array}{l} \text{Chlorophyll}-\text{a }(\text{Ca})=\frac{\left(13.36\text{A}664.2-5.19\text{A}648.6)\times 8.1\right)}{\text{Fresh weight}},\\ \text{Chlorophyll}-\text{b }(\text{Cb})=\frac{\left(27.43\text{A}648.6-8.12\text{A}664.2)\times 8.1\right)}{\text{Fresh weight}},\\ \text{Total carotenoids }(\text{Cx}+\text{c})=\text{C}=\frac{(4.785\text{A}470+3.657\text{A}664.2-12.76\text{A}648.6)\times 8.1}{\text{Fresh weight}},\text{C}\frac{\text{a}}{\text{b}}=\frac{\text{Ca}}{\text{Cb}}\\ \text{Ca}+\text{b}=\frac{(5.24\text{A}664.2+22.24\text{A}648.6)\times 8.1}{\text{Fresh weight}}\text{or Ca}+\text{Cb} \end{array}$$

### Relative Electrolyte Leakage (REL)

Measurement of REL was done according to the method described by Zhang *et al*. (2011). Briefly, 15 freshly cut leaf discs (0.5 cm in diameter) were incubated in tubes with 10 mL of deionised water at room temperature for 6 h. Then the electrical conductivity of the solution (C1) was determined using a conductivity instrument. Further, the tubes were kept in a boiling water bath (100°C) for 25 min and cooled to room temperature, and the total electrical conductivity (C2) was measured. Ion leakage was calculated using the following equation:


$$\text{REL }(\%)=\frac{\text{C}1}{\text{C}2}\times 100$$

### Lipid Peroxidation Activity

Lipid peroxidation activity was estimated by measuring the content of thiobarbutic acid-reactive substances (TBARS) according to Zhang *et al*. (2011). 200 mg of leaf powder was put in 5 mL of methanol and centrifuged at 15000 rpm for 15 min. After that 2 mL sample of supernatant was added to a test tube with 2 mL of the reaction solution containing 20% (w/v) TCA, 0.01% butylated hydroxytoluene and 0.6% thiobarbutic acid. The mixture was heated in boiling water for 15 min and thereafter cooled quickly in an ice bath. After centrifugation at 15000 rpm for 15 min, the absorbance of the supernatant was determined at 450 nm, 532 nm and 600 nm using a UV vis spectrometer. The TBARS content was calculated using the following formula:


$$\text{C}=6.45({\text{OD}}_{532}-{\text{OD}}_{600})-0.56{\text{OD}}_{450}$$

### Measurements of Relative Water Content (RWC)

RWC was measured according to Yang *et al*. (2011). The collected individual leaves were recut from the leaf base with a surgical blade. Thereafter, leaves were immediately weighed (Fresh mass, FM). In order to obtain the turgid mass (TM), leaves were floated in distilled water inside a plastic box for 24 h. At the end of the imbibition period, leaf samples were placed in a hot air oven at 80°C for 48 h in order to obtain the dry mass (DM). Values of FM, TM and DM were used to calculate RWC using the equation:


RWC (%)=[(FM-DM)/(TM-DM)]×100

### Measurement of Leaf Area (LA), Specific Leaf Area (SLA) and Leaf Dry Matter Content (LDMC)

Leaf area (LA) measurement was done according to the graphical method. In this method, a fully expanded leaf of *E. agallocha* was placed on the graph paper. A leaf is drawn with a pencil precisely and carefully on the graph paper. The total number of grids enclosed by the sketch of the leaf was calculated. If the sketch engaged more than one-half grids treated as one, if it employed two-half grids than it is also treated as one otherwise zero. The number of grid count resembles the actual area of a leaf in cm^2^. Immediately after measurement of fresh weight and leaf area, the sample leaves were then kept in a preheated hot air oven at 80°C for two days to get a dry mass of these leaves. Finally, SLA and LDMC were determined according to Garnier *et al*. (2001), using the following equations:


$$\begin{array}{l} \text{SLA}=\frac{\text{Leaf area}}{\text{Leaf dry mass}}\\ \text{LDMC}=\frac{\text{Leaf dry mass}}{\text{Leaf fresh mass}} \end{array}$$

## DATA ANALYSIS

Results obtained in this study were expressed as mean ± standard error of results of three replicates and the student’s *t*-test was applied to evaluate the significance of differences at *p* = 0.05 (Bailey 1995).

## RESULTS

### pH and Electrical Conductivity (EC) of Soil Samples

The pH and EC of the soil have been summarised in Table 2. The present study shows that the soil of Jharkhali and Kolkata were slightly alkaline in nature whereas the soil of Bokkhali showed an acidic nature. Among the three different ecological sites, the Jharkhali soil showed maximum electrical conductivity (2.31 μS/cm) than the other two sites.

### Antibacterial Activity

The antibacterial activity of methanol leaves crude extracts of each sex of *E. agallocha* by agar well diffusion method, which were collected from different ecological sites and their yields have been summarised in Table 2. The yield of extracted compounds for female leaves were 4.2%, 4% and 4% for Jharkhali, Bokkhali and Kolkata samples, respectively, whereas male leaves produced 3.9%, 3.8% and 3.6% for Jharkhali, Bokkhali and Kolkata samples, correspondingly. In addition to this female leaves also showed bacterial inhibition zone against *Bacillus cereus*, *Serratia marcescens, Staphylococcus aureus*, *Salmonella typhi*, *Salmonella enterica*, and *Pseudomonas aeruginosa* were14.1 ± 1 mm, 13 mm, 15 mm, 15 mm, 12 mm and 15 mm, 17 mm, 13 mm, 15 mm, 21 ± 1 mm, 14 mm and 16 mm and 16 mm, 16 ± 2 mm, 15 mm, 21 ± 1 mm, 15 mm and 16 mm for Jharkhali, Bokkhali and Kolkata samples, respectively. The male showed antibacterial activity against *Bacillus cereus*, *Serratia marcescens, Staphylococcus aureus*, *Salmonella typhi*, *Salmonella enterica*, and *Pseudomonas aeruginosa* were 12 ± 1 mm, 10 mm, 14 mm, 14 ± 1 mm, 10 mm and 13 mm, 13 mm, 11 ± 1 mm, 13 mm, 17 ± 1 mm, 11 mm and 12 mm and 16 mm, 16 ± 2 mm, 15 mm, 21 ± 1 mm, 15 mm and 16 mm for Jharkhali, Bokkhali and Kolkata samples, correspondingly. The comparative study showed that in each site the leaf of the female plant yielded much compounds than the male and also the female leaves resulted in higher antibacterial activity against all the selected bacterial pathogens than males.

### Reducing Power

The reducing power of each sex at each site has been summarised in Table 3. From this table it is found that male leaves exhibited reducing power of 39 ± 0 μg, 42 ± 0 μg and 47 ± 0 μg whereas females displayed the values of 48.2 ± 0 μg, 47 ± 0 μg and 54.6 ± 0 μg equivalent of ascorbic acid mg^−1^ of dry weight leaves for Jharkhali, Bokkhali and Kolkata samples, correspondingly. In all cases, the female sex showed the potential of higher reducing power compared to male samples of each site.

### Total Antioxidant Activity

The total antioxidant activity of leaf methanol extracts of each sex of each site of *E. agallocha* by the Phosphomolybdate method has been summarised in Table 3. The total antioxidant activity of male samples were 55.79 *±* 0, 58.79 *±* 0 and 28.63 *±* 0 whereas for females it was 59.79 μg, 107.46 μg and 68.46 μg equivalent to ascorbic acid mg^−1^ of dry weight for Jharkhali, Bokkhali and Kolkata, respectively. Furthermore, The IC_50_ values of females were 22.15 ± 0 μg, 20.5 ± 0 μg and 22.5 ± 0 μg whereas for male samples it were 30.23 ± 0 μg, 29.54 ± 0 μg and 70.14 ± 0 μg equivalent of ascorbic acid for Jharkhali, Bokkhali and Kolkata samples, correspondingly. A lower value of IC_50_ means higher antioxidant activity. From this study, it was also concluded that the female sex was much more effective than the male sex for total antioxidant activity and IC_50_ value (Table 3).

### Chlorophyll-a, chlorophyll-b and Total Carotenoids Estimation

The results of chlorophyll a (Ca), chlorophyll b (Cb), total carotenoids (Cx + c), total chlorophylls (Ca + b) and ratio of chlorophyll-a and chlorophyll-b (Ca/Cb) are displayed in Table 4. The Ca, Cb, (Cx + c) and (Ca + b) of male leaves were 3.81 ± 0, 1.42 ± 0, 0.98 ± 0 and 5.23 mg/g, 3.03 ± 0, 1.7 ± 0, 0.29 ± 0 and 4.73 mg/g, and 3.66 ± 0, 1.23 ± 0, 0.87 ± 0 and 4.9 mg/g fresh weight for Jharkhali, Bokkhali and Kolkata locations correspondingly. On the other side, in the case of females for Jharkhali, Bokkhali and Kolkata samples, these values were 4.92 ± 0, 2.08 ± 0, 1.42 ± 0 and 7 mg/g, 4.98 ± 0, 2 ± 0, 0.75 ± 0 and 6.98 mg/g, and 4.81 ± 0, 1.56 ± 0, 1.04 ± 0 and 6.37 mg/g fresh weight for Ca, Cb, (Cx + c) and (Ca + b), respectively. The results showed that chlorophyll a, chlorophyll b, total carotenoids and total chlorophylls (chlorophyll-a and chlorophyll-b) contents of female leaves of all sites were superior over male leaves whereas the ratio of chlorophyll-a and chlorophyll-b gave the diverse result (Table 4).

### Relative Electrolyte Leakage (REL), Relative Water Content (RWC) and Lipid Peroxidation Activity (TBARS content)

The results of relative water content (RWC), relative electrolyte leakage (REL) and lipid peroxidation activity (TBARS content) are shown in Table 4. The results of these parameters for Jharkhali, Bokkhali and Kolkata male samples were 83.84 ± 1.1, 17.31 ± 0 and 0.18 ± 0 nmol/gm, 89.31 ± 1.6, 18.84 ± 0 and 0.23 ± 0 nmol/gm, and 83.81 ± 1.2, 17.54 ± 0 and 0.46 ± 0 nmol/gm fresh weight for RWC, REL and TBARS content correspondingly. In comparison with this, the female leaves showed the results of RWC, REL and TBARS content were 85.55 ± 1.3, 21.62 ± 0 and 0.44 ± 0 nmol/gm, 87.08 ± 2.2, 28.21 ± 0 and 0.35 ± 0 nmol/gm, and 85.5 ± 1.3, 21.21 ± 0 and 0.65 ± 0 nmol/gm fresh weight for Jharkhali, Bokkhali and Kolkata samples, respectively. The results of this experiment suggested that the percentage of electrolytic leakage of female leaves was higher than male leaves in each site. The higher TBARS contents i.e., higher lipid peroxidation activity in female leaves in each site also indicated the significant findings of electrolytic leakages. However, the relative water contents in leaves gave diverse result.

### Measurement of Leaf Area (LA), Specific Leaf Area (SLA) and Leaf Dry Matter Content (LDMC)

The leaf area of all-male leaves in all sites was found to be greater (34.67 ± 0.33 cm^2^, 36.17 ± 1.88 cm^2^ and 45.67 ± 0.88 cm^2^ for Jharkhali, Bokkhali and Kolkata, respectively) than female leaves (24.33 ± 0.17 cm^2^, 23 ± 0.58 cm^2^ and 29.67 ± 0.67 cm^2^ for Jharkhali, Bokkhali and Kolkata, respectively). However, the specific leaf area and leaf dry matter content gave differing results (Table 4).

### Determination of Total Phenolics

Total phenolics of the leaf methanol extract of each sex of *E. agallocha* have been summarised in Table 4. From this table, it is found that the total phenol content of females were 95.5 ± 0 μg, 132.4 ± 0 μg and 58.6 ± 0 μg equivalent of gallic acid mg^−1^ of dry weight of leaves whereas for males these values were 44 ± 0 μg, 80.8 ± 0 μg and 7.2 ± 0 μg equivalent of gallic acid mg^−1^ of dry weight of leaves for Jharkhali, Bokkhali and Kolkata samples, individually. The results showed that the female sex of each site contains many phenolic compounds rather than the male sex. This finding is related to the results of reducing power, antibacterial activity and total antioxidant activity, where the female sex exhibited much-reducing power, antibacterial activity and total antioxidant activity than males of each site.

### Estimation of Total Protein

The total proteins of leaves of each sex of each site of *E. agallocha* by the Bradford method have been summarised in Table 4. This experiment also showed the richness of total proteins of the female sex (84 ± 0 μg, 79 ± 0 μg and 93 ± 0 μg equivalent of BSA mg^−1^ of fresh weight for Jharkhali, Bokkhali and Kolkata samples, separately) of each site over the male sex (50 ± 0 μg, 63 ± 0 μg and 57 ± 0 μg equivalent of BSA mg^−1^ of fresh weight for Jharkhali, Bokkhali and Kolkata samples, individually).

## DISCUSSIONS

These experimental results showed the differences in biological activities, biochemical constituents, morphological and physiological responses in the natural habituate environment between *E. agallocha* males and females. There are also some notable differences between the species (males and females) of different ecological regions. According to the pH and EC analysis, it assumes that the soil of Jharkhali is richer in various cations and anions than other sites examined in this study. Differences between male species or female species are found because each ecological region has its own climatic condition and several nutrients in the soil. This observation supports the findings of Li *et al*. (2007) that leaf characters are extremely changeable in several environmental gradients in most tree species of dioecious plants.

Plants encounter various enemies in the natural environment by producing a large number and various types of organic compounds (Secondary metabolites), that seem to have no direct purposes in growth and development but have the main role in defence (Majid *et al*. 2011; Kader *et al*. 2020). In this context, the high molecular weight compounds based on carbon rings like phenolic compounds play a tremendous role in the defence. It constitutes 40% or more of the dry mass in leaves (Lincoln 1980; Coley *et al*. 1985; Majid *et al*. 2011). In the present study, the antibacterial activity, reducing power and total antioxidant activity of females were higher than males. The present findings may be related to total phenolic content in female leaves which was higher in females than the males of *E. agallocha* in each site. Certain studies showed that the total phenolic content in crude extract plays a vital role in various biological activities (Tatiya *et al*. 2011; Soare *et al*. 2012; Mak *et al*. 2013; Khaled-Khodja *et al*. 2014). Several studies also indicated that the greater allocations of carbon-based defences are present in the leaves of females as compared to males (Jing & Coley 1990; Espirito-Santo *et al*. 2003).

From this study, it appears that females of *E. agallocha* tolerate more stresses than males as plants can tolerate various stresses by increasing the chlorophyll concentration in leaves (Paul *et al*. 2000; Lattanzio *et al*. 2006). Total protein contents on the leaf also decrease according to the increase in various stresses (Parida *et al*. 2007; Singh *et al*. 2012; Giri *et al*. 2013; Sneha *et al*. 2014). From these results, it might be assumed that females respond more to different stresses than males by increasing the concentration of chlorophylls, carotenoids, and proteins in their leaves.

REL is an indicator of cell membrane damage caused by several environmental stress on plants. The TBARS are the product of lipid peroxidation, which is very effective to measure leaf membrane damage (Zhu *et al*. 2013). This study found that the relative electrolytic leakage and TBARS content were higher in females than males. Recent researches on different environmental stresses on various plants indicate that these stresses increase REL as well as lipid peroxidation activity (Hayat *et al*. 2008; Masoumi *et al*. 2010; Zhu *et al*. 2013; Liu *et al*. 2013). So this finding indicates that various environmental stresses can cause great cellular membrane damage and female plants suffer more serious cell damage than males. From this observation, it may seem that males (Low TBARS content) are superior to females (Comparatively high TBARS content) to tolerate various environmental stresses.

The present study showed that the leaf area of males was higher than females. Hence, it may be concluded that male plants of *E. agallocha* invest more resources in their growth and development than female plants. This is found to be in good agreement with other previous studies, which indicated that females were smaller than males and grow more slowly (Vasiliauskas & Aarssen 1992; Cipollini & Whigham 1994).

The comparisons of this study across sexes showed negative correlations between growth rate and defence. The differences in growth rate (Leaf area) and secondary metabolite content defences between sexes suggest that females invest resource in defences or resistance whereas male invests their resources in growth (Leaf area) or tolerance (Relative electrolytic leakages and TBARS content). This finding agrees with other findings (Obeso 2002; Xu *et al*. 2010) that females of dioecious plant species frequently assign more nutrients for resistance and fewer to growth and maintenance than males. Recently, there has been a spurt in mangrove plantations and management worldwide for ecological restoration using ecological engineering approaches (Hsiang 2000; Amri 2005; Gilman *et al*. 2007). The present study strongly suggested that females are more beneficial according to the use of medicinal properties like antibacterial, reducing power and total antioxidant activities than males due to the presence of higher phenolic compounds in their leaf rather than male sex.

## CONCLUSIONS

*Excoecaria agallocha* females had more biological activities than males. This finding was confirmed by the higher content of various biochemical constituents. From this study, it may concluded that plantation of female of this species are more beneficial than males according to several medicinal properties. The females invest their resources in defences or resistance whereas males invest their resources in growth or tolerance.

## Figures and Tables

**Table 1:** Sample collection sites with their general description, latitude and longitude, soil characteristics and climatic conditions.

Sl. No.	Sites and general description	Latitude and longitude	Soil characteristics	Climatic condition	References
1	Jharkhali The core region of Indian Sundarbans. It is located between the Matla River on the west and the Bidya River in the east.	Latitude 22°01′N and longitude 88°40′E	Semi-solid, medium textured, sandy and silt loam, the grain size variable. Organic matter varies between 3% and 10%.	Mean maximum temperature during Pre-monsoon has been recorded as 32.4°C. Annual relative humidity varies from 70% to 80% and highest during the months of June-October. Annual rainfall is in the range of 1640 mm–2000 mm, maximum rainfall occurs during monsoon from June–October. Experiences tidal storm and wave.	(Sharma 2016)
2	Bakkhali The southernmost point of Indian Sundarbans. There is Hooghly River in the west side and Saptamukhi River in the east side.	Latitude 21°35′N and longitude 88°15′E	The soil is generally sandy in the beach area. However, an inner portion silty soil is found.	During summer (Pre-monsoon) average temperature is 23°C–37°C. Receives maximum rainfall during pre-monsoon. Average annual rainfall is 1600 mm.	(Chattopadhyay & Ghosh 2013)
3	Kolkata The only megacity and capital of the state West Bengal. Located on the eastern region of the Hooghly River. It is the main hub for the industrial, cultural, and educational centre of east as well as northeast India.	Latitude 22°64′N and longitude 88°37′E	The soil is mainly alluvial in origin i.e., loose, unconsolidated soil containing fine particles of silt and clay and larger particles of sand and gravel.	Annual mean temperature is 28.3°C. Pre-monsoon (March–June) are hot and humid. In May and June during dry spells, maximum temperatures frequently exceed 40°C. In April–June thunderstorms namely ‘kal Baisakhi’ bringing cooling support from the existing humidity by heavy rains. Annual rainfall is about 1,640 mm. Average Relative Humidity varies between 47% and 83%.	(Das & Chattopadhyay 2009)

**Table 2:** Comparative antibacterial activities (mm) and extractive yield (%) of male and female plants of *E. agallocha* on methanol leave crude extract, collected from different ecological regions with their soil pH and EC.

Collection sites	Soil pH	Soil EC (μS/cm)	Sex	Yield (%)	Bacterial Strain and diameter of the zone of inhibition (Mean ± Standard error) mm					

					BC	SM	SA	ST	SE	PA
Jharkhali	7.40	2.31	Male	3.9	12 ± 1.00	10 ± 0.00	14 ± 0.58	14 ± 1.00	10 ± 0.00	13 ± 0.58
			Female	4.2	14 ± 1.00	13 ± 0.58	15 ± 0.58	15 ± 0.58	12 ± 0.58	15 ± 0.58
Bokkhali	6.78	0.23	Male	3.8	13 ± 0.58	11 ± 1.00	13 ± 0.00	17 ± 1.50	11 ± 0.00	12 ± 0.58
			Female	4.0	17 ± 0.58	13 ± 0.00	15 ± 0.58	21 ± 1.00	14 ± 0.00	16 ± 0.58
Kolkata	7.12	0.42	Male	3.6	11 ± 0.00	10 ± 0.00	11 ± 2.00	17 ± 1.00	11 ± 0.00	13 ± 0.58
			Female	4.0	16 ± 0.58	16 ± 2.00	15 ± 0.58	21 ± 1.50	15 ± 0.58	16 ± 0.58

*Note*: BC = *Bacillus cereus*, SM = *Serratia marcescens*, SA = *Staphylococcus aureus*, ST = *Salmonella typhi*, SE = *Salmonella enteric*, PA = *Pseudomonas aeruginosa*.

**Table 3:** Comparative studies of reducing power and total antioxidant activities between two sexes of *E. agallocha* collected from different ecological regions.

Biological Activities	Ascorbic Acid (AA)	Sites of collection and plant sex					

		Jharkhali Male	Jharkhali Female	Bokkhali Male	Bokkhali Female	Kolkata Male	Kolkata Female
Reducing power (μg equivalent of AA mg^−1^ of DW)	-	39 ± 0	48.2 ± 0	42 ± 0	47 ± 0	47 ± 0	54.6 ± 0
TAA (μg equivalent of AA mg^−1^ of DW)	-	55.79 ± 0	59.79 ± 0	58.79 ± 0	107.46 ± 0	28.63 ± 0	68.46 ± 0
IC_50_ value of TAA (μg equivalent of AA)	20.63 ± 0	30.23 ± 0	25.15 ± 0	29.54 ± 0	20.50 ± 0	70.14 ± 0	22.5 ± 0

**Table 4:** Morphological and biochemical characteristics of leaves between two sexes of *E. agallocha* collected from different ecological regions.

Leaf parameters	Sites of collection and plant sex (Mean ± Standard error)					

	Jharkhali Male	Jharkhali Female	Bokkhali Male	Bokkhali Female	Kolkata Male	Kolkata Female
Ca (mg/g FW)	3.81 ± 0	4.92 ± 0	3.03 ± 0	4.98 ± 0	3.66 ± 0	4.81 ± 0
Cb (mg/g FW)	1.42 ± 0	2.08 ± 0	1.70 ± 0	2.00 ± 0	1.23 ± 0	1.56 ± 0
Cx + c (mg/g FW)	0.98 ± 0	1.42 ± 0	0.29 ± 0	0.75 ± 0	0.87 ± 0	1.04 ± 0
Ca + b (mg/g FW)	5.23	7.00	4.73	6.98	4.90	6.37
Ca/Cb	2.68	2.37	1.78	2.49	2.98	3.08
REL (%)	17.31 ± 0	21.62 ± 0	18.84 ± 0	28.21 ± 0	17.54 ± 0	21.21 ± 0
RWC (%)	83.84 ± 1.1	85.55 ± 1.3	89.31 ± 1.6	87.08 ± 2.2	83.81 ± 1.2	85.5 ± 1.3
TBARS (nmol/g FW)	0.18 ± 0	0.44 ± 0	0.23 ± 0	0.35 ± 0	0.46 ± 0	0.65 ± 0
LA (cm^2^)	34.67 ± 0.33	24.33 ± 0.17	36.17 ± 1.88	23 ± 0.58	45.67 ± 0.88	29.67 ± 0.67
SLA cm^2^/g	140.92 ± 2.0	186.54 ± 1.1	188.81 ± 9.9	124.26 ± 11.3	256.87 ± 21.3	252.66 ± 6.3
LDMC (mg/g)	0.24 ± 0.01	0.19 ± 0.01	0.20 ± 0.01	0.26 ± 0.01	0.22 ± 0.01	0.21 ± 0.01
Protein (μg equivalent of BSA mg^−1^ of FW)	50 ± 0	84 ± 0	63 ± 0	79 ± 0	57 ± 0	93 ± 0
Phenol (μg equivalent of Gallic Acid mg^−1^ of DW)	44 ± 0	95.5 ± 0	80.8 ± 0	132.4 ± 0	7.2 ± 0	58.6 ± 0
